# Microstructural and Elemental Characterization of Calcium Silicate-Based Sealers

**DOI:** 10.3390/nano15100756

**Published:** 2025-05-18

**Authors:** Mateusz Radwanski, Ireneusz Piwonski, Tomasz Szmechtyk, Salvatore Sauro, Monika Lukomska-Szymanska

**Affiliations:** 1 Department of Endodontics, Medical University of Lodz, 251 Pomorska Str., 92-213 Lodz, Poland; mateusz.radwanski@umed.lodz.pl; 2 Department of Materials Technology and Chemistry, Faculty of Chemistry, University of Lodz, 163 Pomorska St., 90-236 Lodz, Poland; ireneusz.piwonski@chemia.uni.lodz.pl; 3 Department of Physical Chemistry, Faculty of Chemistry, University of Lodz, 163/165 Pomorska St., 90-236 Lodz, Poland; tomasz.szmechtyk@chemia.uni.lodz.pl; 4 Dental Biomaterials and Minimally Invasive Dentistry, Departamento de Odontología, Facultad de Ciencias de la Salud, Universidad CEU-Cardenal Herrera, C/Del Pozo ss/n, 46115 Alfara del Patriarca, Valencia, Spain; salvatore.sauro@uch.ceu.es; 5 Department of Therapeutic Dentistry, I.M. Sechenov First Moscow State Medical University, 119146 Moscow, Russia; 6 Department of General Dentistry, Medical University of Lodz, 251 Pomorska Str., 92-213 Lodz, Poland

**Keywords:** calcium silicate-based sealers, EDX, root canal sealer, SEM, XRD

## Abstract

Calcium silicate-based sealers (CSBS) vary in chemical composition, which can influence treatment outcomes. Therefore, the study aimed at comparing several commercially available CSBS regarding microstructure and elemental characterization. Four CSBS (AH Plus Bioceramic Sealer, BioRoot RCS, BioRoot Flow, TotalFill BC Sealer) and a control resin-based sealer (AH Plus) were evaluated using scanning electron microscopy (SEM), energy-dispersive X-ray spectroscopy (EDX), and X-ray powder diffraction analysis (XRD). The specimens were analyzed after setting (SEM, EDX, XRD), as well as after 7 (SEM) and 28 days (SEM, EDX) of incubation in Hank’s balanced salt solution. AH Plus exhibited a uniform matrix and small amounts of calcium (Ca), significantly decreasing after incubation. In contrast, CSBSs exhibited crystalline forms on the surface and increased Ca content, significantly increasing after 28 days of incubation. The main crystalline phase for all tested CSBS was zirconium oxide, while for ERBS it was calcium tungstate. In conclusion, the amount of calcium increased on the surface of CSBSs after incubation, which alkalinized the pH, promoting mineralization, apatite formation, and antibacterial potential. Despite this, the formation of a hydroxyapatite layer was not demonstrated, possibly due to the high dissolution potential of CSBSs.

## 1. Introduction

Root canal filling plays a key role in long-term endodontic treatment success. Fluid-tight seal obturation, especially of the apical part, prevents the development of pathological processes or ensures proper healing of existing lesions in the periapical tissues [1]. To provide adequate obturation, gutta-percha and sealer are used [2]. The role of the sealer is to fill the space between the gutta-percha and the canal walls, seal places that are difficult to access with instruments, i.e., lateral canals and irregularities, and also to connect gutta-percha cones when the condensation technique is applied [3].

Many sealers are available on the market, differing in their chemical composition and binding reaction. Considering the composition, the following groups of sealants can be distinguished: zinc oxide and eugenol, calcium hydroxide, glass ionomer, epoxy resins, silicone, and calcium silicates (hydraulic/bioceramic) [4].

Epoxy resin-based sealers (ERBS, e.g., AH Plus) are a group of hydrophobic sealers that are characterized by small volumetric changes over time, antibacterial properties, and high radiopacity [5]. AH Plus is widely applied in everyday practice and has proven long-term clinical success, so it is considered the “gold standard” [6,7]. On the other hand, this material exhibits certain disadvantages of ERBS, such as difficulty in connection with gutta-percha [8], altered setting in the presence of moisture [9], potential cytotoxicity and genotoxicity [10], and complications during removal in retreatment [5].

Recently, calcium silicate-based sealers (CSBS) have become increasingly popular. CSBS requires water for hydration reaction. Although they are called bioceramics, the term is not specific. The term “hydraulic” is more adequate because it describes the interaction with water aiming at hydration, as well as the need for water after setting to develop the sealer properties [11].

The initial reaction of CSBS with water produces calcium silicate hydrate and calcium hydroxide. Calcium and hydroxyl ions are released, which ensures a high pH (>12) and thus good antibacterial activity. Additionally, alkaline pH is maintained for a long time, promoting long-term elimination of bacteria [12,13].

Tissue fluids and calcium ions released from the sealer are necessary for depositing hydroxyapatite (HA) crystals on the dentin surface. As a consequence, a hydroxyapatite mineral layer enhances the bond between the sealer and the tooth [12,14,15].

CSBS, including MTA Fillapex and EndoSequence BC Sealer, are recognized for their favorable physical properties, which encompass calcium ion release and flowability. Nonetheless, their elevated solubility continues to pose a considerable challenge. Research has indicated that although these sealers demonstrate commendable performance in terms of film thickness and calcium ion release, their solubility may compromise their long-term stability and sealing efficacy [16].

HA formation in CSBS is essential for their biomineralization potential and effectiveness in root canal treatments. Sealers like Ceraseal and AH Plus Bioceramic induce hydroxyapatite precipitation on root dentin by releasing calcium ions, which interact with phosphate ions in tissue fluids [17]. Several factors influence HA formation, primarily the pH level; higher pH enhances precipitation, with Ceraseal showing greater alkalizing activity. The sealers’ hydration process also matters; faster setting can reduce flowability but increase HA formation rates. Immersion in phosphate-buffered saline (PBS) aids HA deposition on dentin, which is often used in studies to assess sealers’ biomineralization potential [18]. The capability to form HA is vital for effectiveness of the seals, as it prevents micro-leakage and promotes healing of periapical tissues, enhancing both physical properties and biocompatibility, making these sealers preferred in endodontic treatments.

However, amorphous calcium silicate hydrates (C-S-H) are typically characterized using spectroscopic techniques such as Micro-Raman, Fourier Transformed Infrared (FT-IR), and Nuclear Magnetic Resonance (NMR). These techniques help identify the structural and chemical properties of C-S-H, which is essential for understanding its behavior in various applications [19].

CSBS can be defined as bioactive, positively influencing cellular interactions [20] and promoting osteoblast differentiation and deposition of cells necessary for wound healing [21]. The main disadvantages of sealers include increased solubility and resorption of the material over time. Moreover, unfavorable interactions with irrigation agents (e.g., citric acid, EDTA) were observed [22,23]. It can be explained by the fact that a pH below 8.8 causes the loss of stability of hydrated calcium silicate formed by the hydration reaction. This phenomenon, in turn, accelerates the degradation process of the material by dissolving calcium-containing compounds [24]. In addition, calcium ions react with acid to form salts that, if soluble, are washed out of the material, creating pores. In contrast, the insoluble salts can cause material expansion and crack propagation [25]. This can cause voids and material resorption, leading to bacterial microleakage. Conversely, these agents can dissolve CSBS materials during retreatment.

Moreover, CSBS is available in a wide range of formulations. Some are produced as two mixed pastes, while others are available as powder and liquid, requiring mixing to obtain a paste (e.g., BioRoot RCS). A separate group consists of sealers in the form of a single paste, premixed, that can be applied to the root canal directly from a syringe using an appropriate cannula (e.g., AH Plus Bioceramic Sealer, BioRoot Flow, TotalFill BC Sealer) [26].

Many sealers with different chemical compositions are classified as CSBS, but they have other properties; this may affect the structures of the sealer, as well as the quality and outcome of root canal treatment [27,28]. Differences in the composition of the material can contribute to variable ionic interactions with the surrounding tissues and trigger a different cellular response [29]. Released substances on the surface of the material can cause the formation and deposition of compounds that will increase the bonding of the sealer to the root dentine [30]. Moreover, the release of some particles from these materials can be potentially toxic, as when in contact with tissues, these can cause irritation, inflammation, and possibly a delay in healing [31].

Thus, this study aimed to assess the microstructure and chemical properties of four CSBS and compare them with the gold-standard sealer, ERBS.

The null hypothesis tested in this study was that there would be no difference in microstructure and chemical properties of the tested materials between different observation and measurement points.

## 2. Materials and Methods

**Figure 1:**
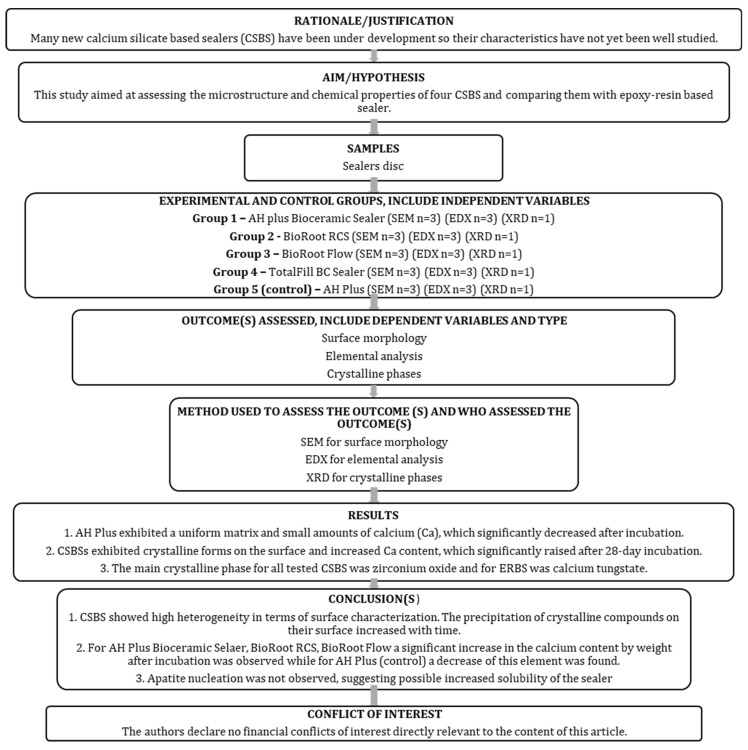
The PRILE 2021 flowchart of this study.

The manuscript of this laboratory study has been written according to the Preferred Reporting Items for Laboratory studies in Endodontology (PRILE) 2021 guidelines [32,33]. Figure 1 shows the PRILE 2021 flowchart, and the PRILE 2021 checklist is attached to Supplementary Files (Table S1).

### 2.1. Root Canal Sealers

In the present study, four calcium-silicate-based sealers: AH Plus Bioceramic Sealer, BioRoot RCS, BioRoot Flow, TotalFill BC Sealer, and epoxy-resin-based: AH Plus were analyzed. Their characteristics are presented in Table 1.

### 2.2. Sample Preparation

Silicon molds (10 mm diameter and 2 mm thick) were prepared and used in the current study to set the root canal sealer materials. The BioRoot RCS and AH Plus were mixed according to the manufacturer’s instructions; other materials were premixed and ready to use. The sealers were allowed to set in a laboratory thermostat at 37 °C and 95% relative humidity.

### 2.3. SEM Imaging

Before imaging, all specimens were sputter-coated with 20 nm of gold for electrical conductivity and then examined using a scanning electron microscope (SEM, FEI Nova NanoSEM 450, FEI, Hillsboro, OR, USA), with an accelerating voltage of 5 kV, WD = 4.8 ± 0.2 mm. The representative SEM images for each study group were captured at 1000×, 2000×, 5000×, and 10,000× magnification for the characterization of the specific microstructure. The assessment was carried out directly after setting, and after 7- and 28-day incubation in Hank’s balanced salt solution (HBSS; PA-36-3057-H; Pol-Aura, Poland). The samples were immersed upright in 20 mL of HBSS, and the medium was replaced weekly. All tests were conducted in triplicate.

### 2.4. EDX Analysis

For elemental analysis, specimens were examined with an energy-dispersive spectrometer (EDX, EDAX/AMETEK, Materials Analysis Division, Model Octane Super, Mahwah, NJ, USA), using an accelerating voltage of 10 kV. After removing specimens from silicon molds, the EDX spectra were performed in the middle region of each specimen in three different locations. Elements without previous selection that appeared in the spectrum were analyzed. The specimens were placed directly onto the SEM stub and examined without any previous coating procedures. The surfaces were analyzed after setting up and after 28 days of incubation in HBBS. The specimens were immersed upright in 20 mL of HBSS, and the medium was replaced weekly.

### 2.5. XRD Investigation

The crystalline structure and chemical composition were investigated by an X-ray powder diffraction analysis (XRD) system (Aeris 1.2.0 X-ray diffractometer, Malvern Panalytical, Malvern, Worcestershire, UK) with PIXcel1D-Medipix3 detector and Cu radiation (CuK_α_ *λ* = 1.54178 Å). After setting, the disc specimens were ground progressively till a finer powder was obtained. The powder was placed in a back-loaded sample holder and pressed. The test was conducted in continuous mode at an angle 2Θ range of (15–60°) with a scanning rate of 0.9°/s under 7.5 mA at 40.0 kV. The attained XRD patterns were interpreted using the model patterns on the ICDD database (International Centre for Diffraction Data, Newtown Square, PA, USA) and COD database (Crystallography Open Database).

### 2.6. Statistical Analysis

The Shapiro–Wilk test was used to confirm the normality of the data. Scheffé test (post-hoc) was used to compare repeated measurements of ion changes over time (EDX) in tested materials. All statistical analyses were evaluated with the statistical software package Statistica v. 13.1 (StatSoft, Inc., Tulsa, OK, USA), and statistical significance was considered at *p* < 0.05.

## 3. Results

### 3.1. SEM Imaging

**Figure 2:**
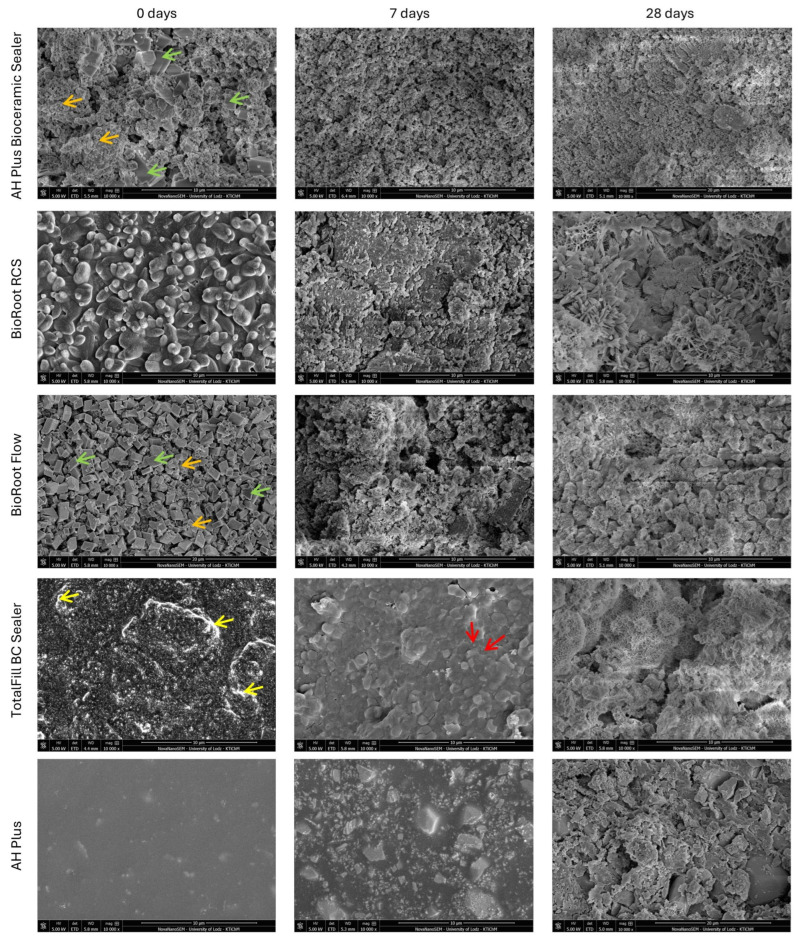
Representative SEM images of materials morphology after setting (0 days), 7-, and 28-day (10,000×) incubation periods. In AH Plus Bioceramic Sealer and BioRoot Flow, orange arrows indicate spherical particles, and green arrows indicate cuboidal particles. For TotalFill BC Sealer, yellow arrows indicate the particles rich in zirconium, and red arrows indicate the hydration rims.

Representative SEM images of materials morphology after setting (0 days), 7-day, and 28-day (10,000×) incubation periods were presented in Figure 2.

In the case of the AH Plus Bioceramic Sealer, the baseline SEM analysis showed different shapes of the powder particles; predominantly smaller spherical with larger cuboidal. After 7 and 28 days of incubation in HBSS, granules and small crystals covered the material’s surface.

SEM images of BioRoot RCS revealed homogenous spherical particles in different sizes. The 7-day immersion in HBSS induced the precipitation of a layer of globular crystallites on the material surface. The SEM analysis after 28 days showed the development of the crystal lattice on the surface of the tested material.

BioRoot Flow was characterized by the presence of cuboid particles with a small number of round incorporations. The immersion in HBSS, both after 7 days and 28 days, induced the precipitation of a layer of crystallites on the material’s surface.

SEM of TotalFill BC Sealer disclosed large amounts of zircon-rich areas in the material. After 7 days, the TotalFill BC Sealer exhibited a high degree of hydration, which was confirmed by the presence of reaction rims. After 28 days, the material was covered with precipitated crystals.

SEM microphotographs of AH Plus revealed an uneven surface of granular particles with varying sizes. After 7 days, the specimens showed a slightly rough surface. Moreover, after 28 days of incubation, the structure of the material became inhomogeneous, with the presence of voids.

### 3.2. EDX Analysis

**Figure 3:**
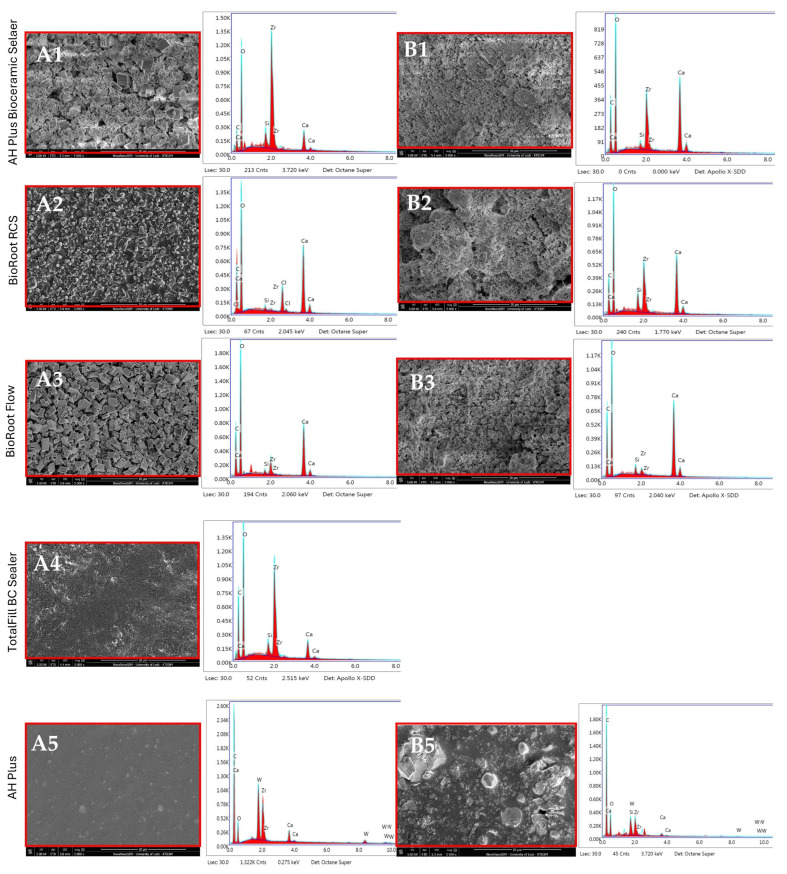
Scanning electron microscopic (SEM) images (5000×) and EDX results of tested materials after (**A**) setting (0 days) and (**B**) 28 days incubation in HBSS. Red rectangles indicate the areas for elemental analysis.

EDX elemental analysis of AH Plus Bioceramic Sealer revealed predominantly Zr (zirconium), O (oxygen), Ca (calcium), C (carbon), and slight traces of Si (silicon). After 28 days, the surface was characterized by a statistically higher presence of Ca (31.86% wt.), O (38.95% wt.), and C (12.18% wt.) and a significant decrease in Zr and Si content (Figure 3, Table 2).

Table 2 compares elements detected on the surface of tested materials (% weight) after setting (0 days) and a 28-day incubation period.

After setting, the surface of BioRoot RCS contained mainly O and Ca. Moreover, the elemental analysis showed Zr, C, Cl (Chlorine), and Si. Subsequent to 28 days in HBSS, the surface was characterized by a higher presence of Ca, Zr, and Si and a slight decrease in C and O. However, Cl was not detected (Figure 3, Table 2). The most significant alterations in composition were observed for the following elements: O (decrease) and Ca, Zr, Si (increase) (*p* < 0.05).

After setting, the main elements detected on the surface of the BioRoot Flow were O (43.47% wt.) and Ca (36.75% wt.). Additionally, Zr, C, and Si were found. After 28 days, significant alterations in composition were observed for C, Si, Ca (increase), and for Zr (decrease) (*p* < 0.05) (Figure 3, Table 2). The elemental analysis of TotalFill BC Sealer revealed that the surface was mainly characterized by the presence of O, Zr, and C, and slight traces of Ca and Si. The 28-day analysis could not be undertaken as the specimens degraded on immersion in HBSS (Figure 3, Table 2). The surface of AH Plus before incubation mainly contained C (70.52% wt.) and O (21.64% wt.) and small amounts of Zr, W (Tungsten), and Zr. After incubation for 28 days, the Si were additionally detected. At the end point measurements, the most significant changes in composition were an increase in O and a decrease in Zr, Ca, and W content (*p* < 0.05) (Figure 3, Table 2).

### 3.3. XRD Investigation

**Figure 4:**
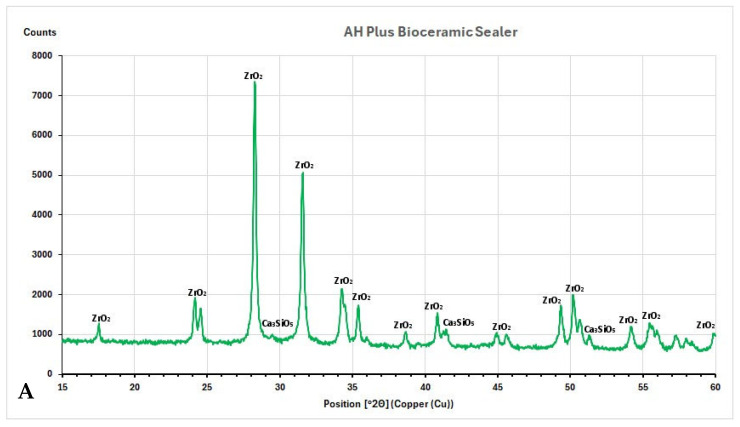

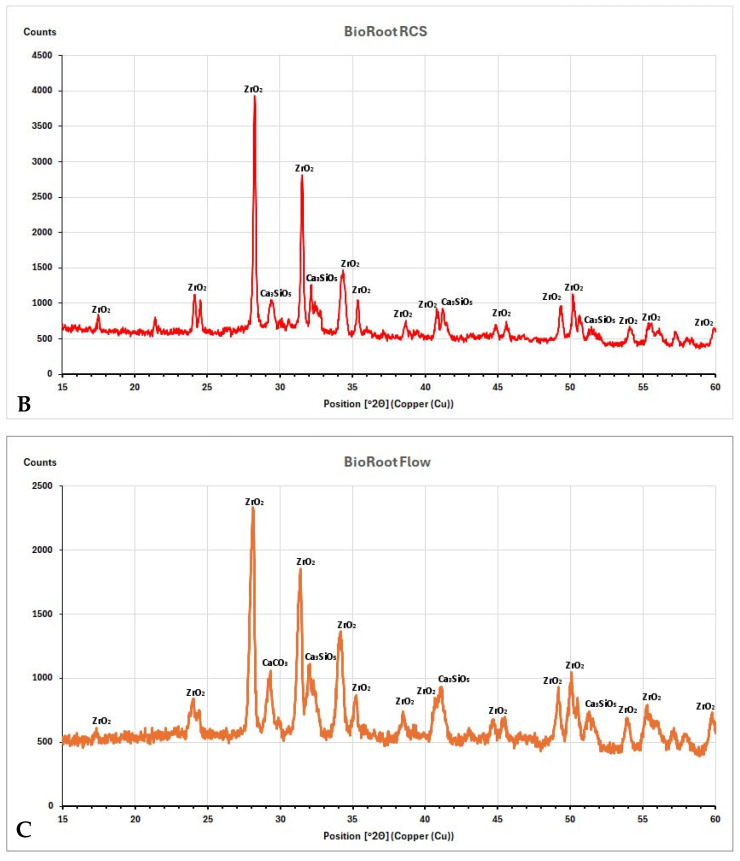

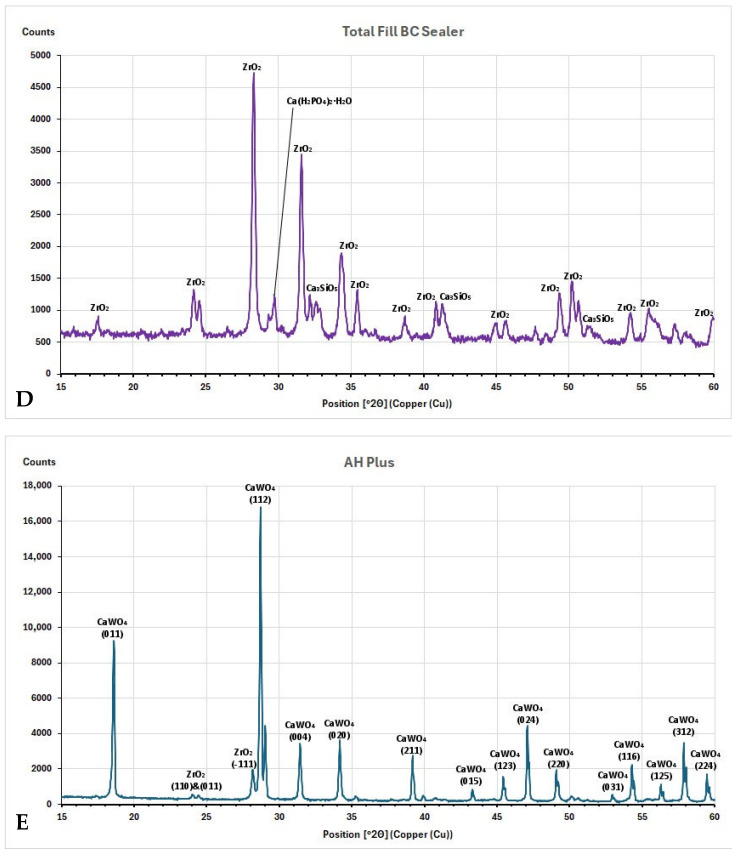
(**A**–**E**)**.** X-ray diffraction plots of all sealers tested immediately after setting show the main phases.

The X-ray diffraction (XRD) plots with identified peaks are shown in Figure 4A–E.

The XRD analysis showed that the AH Plus Bioceramic Sealer contained zirconium oxide (ZrO_2_, JCPDS 96-230-0297) and tricalcium silicate (Ca_3_SiO_5_, alite, COD 96-901-6126) (Figure 4A). Similarly, BioRoot RCS showed peaks of zirconium oxide (ZrO_2,_ JCPDS 96-230-0297) and tricalcium silicate (Ca_3_SiO_5_, alite, COD 96-901-6126) (Figure 4B). For both materials, most of the planes of the aforementioned sealers correspond with monoclinic ZrO_2_, with most prominent peaks at 2Θ = 28.25 and 2Θ = 31.53. In BioRoot Flow, instead of zirconium oxide (ZrO_2_, COD 96-900-7449) and tricalcium silicate (Ca_3_SiO_5_, alite, COD 96-901-6126), the peak of calcium carbonate (CaCO_3_, calcite, JCPDS 47-1743) was detected (Figure 4C). This peak (2Θ = 29.26) corresponds with the (104) plane. TotalFill BC Sealer was composed of zirconium oxide (ZrO_2_, JCPDS 96-230-0297), tricalcium silicate (Ca_3_SiO_5_, alite, COD 96-901-6126), and the reaction product of calcium phosphate monobasic (Ca(H_2_PO_4_)_2_ * H_2_O) with ICDD 00-002-0647 (Figure 4D). The AH Plus included calcium tungstate (CaWO_4_, JCPDS 96-900-9628) and zirconium oxide (ZrO_2_, JCPDS 96-230-0297) (Figure 4E). Almost all AH Plus sealer planes belong to CaWO_4_, with the two most prominent peaks at 2Θ = 18.57 (011) and 2Θ = 28.69 (112).

## 4. Discussion

Despite continuous development, no ideal endodontic sealer is available that meets all demands. In vitro studies contribute to the understanding of the properties of sealers for clinical applications. In addition, the chemical composition of sealers determines their features, including pH, flow, dimensional changes, radiopacity, and working time. The mentioned characteristics influence the formation of a hermetic seal, thus promoting the healing of periapical tissues. The present study tested the microstructural and elemental characterization of four CSBS and one ERBS using SEM, EDX, and XRD analysis. The null hypothesis was rejected because significant differences were found in the microstructure and chemical properties of the tested materials between different observation and measurement points.

### 4.1. Characterization of Root Canal Sealers Through SEM

SEM evaluation is valuable for assessing the surface morphology and texture of materials. As far as CSBSs are concerned, they can also be applied to determine their hydration [34,35]. The degree of cement hydration can affect properties such as mechanical strength, volume stability, and durability [36]. In this study, the analysis of SEM images showed a crystalline surface of all CSBSs, in contrast to the epoxy resin sealer being a uniform matrix with interposition of different-sized particles. Interestingly, each of the CSBSs exhibited a different crystalline type; the specimens were heterogeneous, which was confirmed by the results of other studies [37,38,39,40]. Additionally, hydration was detected in the SEM image of TotalFill BC Sealer, which was supported by others [38]. The material was characterized by finer particles and a more densely packed structure, providing a large surface area facilitating cement hydration.

### 4.2. Characterization of Root Canal Sealers Through EDX

At the baseline (0 days), EDX results in CSBS sealers showed various proportions of oxygen, zirconium, carbon, and calcium.

The highest amount of Zr was found in the AH Plus Bioceramic Sealer (45.48% wt.), which was confirmed by others and by the manufacturer (50–75% zirconium dioxide) [38]. In the case of TotalFill BC Sealer, the Zr content was also high (32.86% wt.), which corresponded to the composition given by the manufacturer (zirconium oxide—35–45%) [38].

Different radiopacifiers may be added to the sealer formulation to differentiate material introduced into the canal from nearby anatomical structures, thus facilitating the assessment of canal filling quality during radiographic examination. In the present study, the tested CSBS revealed the presence of zirconium and oxygen, which was related to the presence of zirconium oxide as a contrasting substance. Zirconium oxide is a biocompatible compound that does not cause tooth discoloration [39,41,42]. Moreover, it provides adequate radiopacity and does not interfere with the hydration of CSBS (when added in quantities less than 30%), thus not affecting the setting reaction [43]. However, if the radiopacifier content is increased, the proportions of the main active ingredients must be reduced. This may result in a deterioration of material properties, such as compressive strength and push-out strength [44,45].

Additionally, in the present EDX analysis, all tested sealers (CSBSs and ERBS) presented heavy metals such as bismuth, lead, chromium, cobalt, copper, zinc, or manganese below the detection limit. These metals are toxic and can induce tooth discoloration [39,43,46].

Another important element detected in EDX analysis for all evaluated CSBS was calcium. The release of calcium plays an active role in apical tissue repair (increasing pH, providing an antibacterial effect) and is also responsible for the formation of calcite crystals, which directly contributes to the development of the mineralized barrier [47]. In the present study, BioRoot RCS and BioRoot Flow exhibited the highest calcium contents, consistent with previous studies [48,49]. BioRoot Flow included calcium hydroxide and calcium carbonate alongside tricalcium silicate, whereas BioRoot RCS incorporates calcium chloride. The differences in calcium content may result from the higher release of this ion from the sealers [50].

In the case of BioRoot RCS, EDX showed additionally the presence of chlorine (Cl), a component of the vehicle (aqueous solution of calcium chloride), which was also observed by others [4,51]. In contrast to one study [4], the present analysis did not reveal the peaks for nitrogen (N), a component of polycarboxylate in BioRoot RCS. This discrepancy could be explained by the fact that C and N had similar locations in the EDX spectrum, and consequently, N might not have been detected due to peak overlap.

Moreover, BioRoot RCS and BioRoot Flow showed similar elemental compositions, except for Cl for BioRoot RCS, which was confirmed in another study [40]. Interestingly, these materials differ in composition, both in terms of the vehicle (BioRoot RCS—water and BioRoot Flow—propylene glycol) and the number of additives in the case of BioRoot Flow (e.g., acrylamide, isohexadecane, polysorbate).

AH Plus comprised C and O, with small amounts of Zr and Ca. In the present study, a slight trace (2.38% wt.) of tungsten (W) was detected and confirmed by another study [52]. Conversely, others reported a higher incidence of W in tested AH Plus samples (approximately 35%) compared to the present results [34,53].

Furthermore, the EDX analysis was performed after incubation to determine the nucleation of calcium phosphates induced in simulated body fluid. For this purpose, an HBSS solution devoid of calcium and magnesium ions was used as the incubation medium. The exclusion of ions was because the presence of additional magnesium and calcium ions can influence the structure of calcium silicate hydrate and its stability [40,54]. Moreover, some studies confirmed that incubation in a medium containing these ions could distort their content in the specimens after incubation, overestimating their authentic chemical behavior [37,40].

It has been proven that materials based on calcium silicates, when put in contact with fluids containing phosphates, were able to produce crystals resembling apatite or its precursors [55]. The hydroxyapatite created in this way increases the tightness of the filling material and promotes the adhesion of cells forming hard tissues to the surface of the material by adsorbing fibronectin, causing the formation of a biological barrier, thus preventing bacterial infiltration [56].

CSBS, after immersion in HBSS, analyses showed a layer rich in Ca and C, without phosphorus (P) peaks. Additionally, the nucleation capacity of apatite was low. A possible explanation could be attributed to the overlap of Zr and P peaks in EDX, which might mask the presence of P on their surface [57]. Therefore, the high content of Zr in the material (e.g., AH Plus Bioceramic Sealer, ca 50–75% ZrO_2_) may distort the measured amount of P, resulting in the underestimation of the latter [37].

Additionally, the lack of apatite crystallization on the tested sealers may be attributed to carbonation processes, which take place during the setting reaction [38]. The released calcium ions react with carbonate ions, forming a surface layer of calcium carbonate, most often in the form of calcite (CaCO_3_) [58]. CaCO_3_ precipitates both on the surface and in the porosities of the sealer, forming a protective layer, limiting the diffusion of ions, and reducing its degradation and/or solubility, thus improving the sealing [59].

The low crystallization capacity of apatite in the case of AH Plus Bioceramic Sealer may be related to the low content (15%) of calcium silicate in its composition. A lower content indicates fewer silanol (Si-OH) functional groups, which are necessary for apatite nucleation [60,61]. In the case of BioRoot RCS and BioRoot Flow, the silicate content is not provided by the manufacturer, therefore, a direct comparison is impossible. In the present study, it is not plausible to discuss the results of nucleation of CaP in the case of TotalFill BC Sealer due to the degradation of the specimens after immersion. However, studies showed significantly higher nucleation activity of TotalFill BC Sealer samples due to the content of monobasic calcium phosphate in the composition and a higher content of tricalcium and dicalcium silicates (tricalcium silicate: 20–35% and dicalcium silicate: 7–15%) [37,62].

AH Plus did not show any nucleation, which was consistent with previous studies that found no calcium phosphate deposit on AH Plus after immersion in HBSS [37,48,63].

However, EDX showed the distribution of elements from several micrometers below the sample’s surface. Therefore, other complementary techniques, such as X-ray diffraction (XRD) analysis, are needed to identify sealer crystalline phases after setting.

### 4.3. Characterization of Root Canal Sealers Through XRD

The main phase identified by XRD for CSBS was the same—zirconium oxide, which was confirmed by other studies [30,38,40]. It should be emphasized that the main component of any commercially available CSBS material is usually calcium silicate. The lack of calcium silicate peaks in XRD may be due to the detection limits for amorphous structures and the overlap of some peaks in the multiphase material [40]. Nevertheless, the predominance of the zirconium oxide phase corresponds to the fact that the amount of zirconium oxide in these materials is usually greater than that of calcium silicate [40].

AH Plus was mainly composed of calcium tungstate (Ca(WO_4_)) with two peaks that represented zirconium oxide (ZrO_2_). The obtained results were consistent with previous studies [51,64].

### 4.4. Limitations of the Study

Some limitations of the present study should be acknowledged. The study is in vitro and provides only a preclinical evaluation of the sealers. The silicone molds were used to prepare the samples. Using an in vitro model does not reproduce the actual conditions in the root canal, i.e., anatomical complexity and interaction with dentin. Thus, further studies should be performed in in vivo models (e.g., animal-based models) [65,66] or under clinical conditions. The incubation time of the specimens was 28 days. However, it seems reasonable to require a longer observation period to assess the long-term stability of the material and structural changes. Additionally, detecting apatite crystals in in vitro models to evaluate bioactivity is still questionable and does not include the role of environmental factors such as temperature, pH, carbon dioxide partial pressure, and agitation. Also, manual mixing of one sealer (BioRoot RCS) introduced an operator-dependent factor that might have affected the outcomes. The research does not monitor chemical changes before moisture adsorption; therefore, investigations of the physicochemical properties of freshly mixed and set root canal sealers should be conducted. Moreover, further studies should be supplemented with elemental chemical analysis using X-ray fluorescence (XRF). In addition, it is recommended that chemical changes be investigated before, during, and after heating using Fourier Transform Infrared Spectroscopy (FTIR), considering the time of heat application.

### 4.5. Summary and Future Perspectives

The present study confirmed that despite classifying CSBS materials into the same group of sealers, they differed in surface structure, elemental composition, and crystalline phases. This discrepancy might affect their clinical use and treatment outcomes. Moreover, CSBS demonstrated the ability to release biologically relevant ions (calcium), but despite this, after incubation in body-simulated fluids, a hydroxyapatite layer was not formed on their surface. It is worth emphasizing that laboratory tests are essential and necessary to determine the properties of the materials and assess their suitability for clinical applications. Due to the high heterogeneity of CSBS materials, further studies must be performed to determine the extended effect of different formulations of CSBS on treatment outcome.

## 5. Conclusions

Within the limitations of the study, it can be stated that:

1. Despite being classified in the same group, calcium-silicate-based sealers showed high heterogeneity in terms of surface characterization both immediately after setting and during the incubation period. The precipitation of crystalline compounds on their surface increased with time.
2. For AH Plus Bioceramic Selaer, BioRoot RCS, and BioRoot Flow, a significant increase in the calcium content by weight after incubation was observed, while for AH Plus (control), a decrease in this element was found. Consequently, it can be hypothesized that the increase in pH may induce mineralization, apatite formation, and antibacterial potential, promoting healing.
3. Apatite nucleation was not observed, suggesting possible increased solubility of the sealer. Consequently, a compromised seal in the apical region could lead to clinical failure.
4. The main crystalline phase for all tested CSBS was zirconium oxide, and for ERBS was calcium tungstate.

## Figures and Tables

**Table 1:** Materials used in this study.

Root Canal Sealer	Manufacturer	Composition	LOT
AH Plus Bioceramic Sealer	Manufactured by Maruchi Distributed by Denstply DeTrey GmbH Konstanz, Germany	Zirconium dioxide (50–75%), tricalcium silicate (5–15%), dimethyl sulfoxide (10–30%), lithium carbonate (<0.5%), thickening agent.	KI211103
BioRoot RCS	Septodont, Saint Maur Des Fosses, France	Powder: Tricalcium silicate, zirconium oxide, and povidone. Liquid: Aqueous solution of calcium chloride and polycarboxylate.	B30546
BioRoot Flow	Septodont, Saint Maur Des Fosses, France	Tricalcium silicate, propylene glycol, povidone, calcium carbonate, aerosil (silica), zirconium oxide, acrylamide/sodium acryloyldimethyltaurate copolymer, isohexadecane and polysorbate.	B33990AAB
Total Fill BC Sealer	FKG Dentaire, La Chaux-de-Fonds, Switzerland	Calcium silicates, calcium phosphate monobasic, zirconium oxide, tantalum oxide, and thickening agents.	24003SP
AH Plus	Denstply DeTrey GmbH, Konstanz, Germany	Paste A: bisphenol-A epoxy resin, bisphenol-F epoxy resin, calcium tungstate, zirconium oxide, iron oxide, pigments. Paste B: dibenyldiamine, aminoadamantane, tricyclodecane-diamine, calcium tungstate, zirconium oxide, silica, silicone oil.	2207000296

**Table 2:** Comparison of elemental analysis (% weight) of all tested materials after setting and 28 days incubation. Significant *p* < 0.05 increase (↑) and decrease (↓) during the incubation period were marked.

Element	AH Plus Bioceramic Sealer		BioRoot RCS		BioRoot Flow		TotalFill BC Sealer		AH Plus	
	0 d	28 d	0 d	28 d	0 d	28 d	0d	28 d	0 d	28 d
C	8.56	12.18 ↑	7.99	7.94	10.71	14.16 ↑	22.28	ND	70.52	71.47
O	30.74	38.95 ↑	40.74	37.06 ↓	43.74	44.15	32.99	ND	21.64	24.94 ↑
Si	2.57	0.77 ↓	0.80	2.78 ↑	0.76	1.22 ↑	1.92	ND	ND	0.66
Zr	45.48	16.24 ↓	10.91	17.84 ↑	8.04	2.06 ↓	32.86	ND	4.37	2.49 ↓
Cl	ND	ND	7.56	ND	ND	ND	ND	ND	ND	ND
Ca	12.65	31.86 ↑	32.00	34.38 ↑	36.75	38.41 ↑	9.94	ND	1.09	0.26 ↓
W	ND	ND	ND	ND	ND	ND	ND	ND	2.38	0.17 ↓

Carbon (C); oxygen (O); silicon (Si); zirconium (Zr); chloride (Cl); calcium (Ca); tungsten (W); not detectable (ND).

## Data Availability

Data are contained within the article and Supplementary Materials.

## Supplementary Materials

The following supporting information can be downloaded at: https://www.mdpi.com/article/10.3390/nano15100756/s1. Table S1: PRILE 2021 checklist.
